# Effects of an examiner’s positive and negative feedback on self-assessment of skill performance, emotional response, and self-efficacy in Korea: a quasi-experimental study

**DOI:** 10.1186/s12909-019-1595-x

**Published:** 2019-05-14

**Authors:** Eun Jung Kim, Kyeong Ryong Lee

**Affiliations:** 1 School of Nursing, Research Institute of Nursing Science, Hallym University, 1 Hallymdaehak-gil, Chuncheon, Gangwon-do 24252 Republic of Korea; 2 Department of Emergency Medicine, School of Medicine, Konkuk University, Konkuk University Medical Center, 120 Neungdong-ro, Gwangjin-gu, Seoul, 05029 Republic of Korea

**Keywords:** Feedback, Self-efficacy, Task performance and analysis, Nursing education

## Abstract

**Background:**

Feedback is an essential element in performance training. However, little effort has been made to measure the effects of positive and negative feedback on the ability of self-rated assessment, affective responses, and motivation to learn in healthcare education.

**Methods:**

This study was a quasi-experimental posttest design to examine the effects of an examiner’s positive and negative verbal feedback on the accuracy of self-assessment, emotional responses, and self-efficacy. Second-year nursing students were recruited in a university in South Korea. A total of 110 participants were assigned randomly to a positive feedback (PF) group (*n* = 58) and a negative feedback (NF) group (*n* = 52). All participants completed the performance measure and then received a positive or negative feedback from an evaluator. After delivery of feedback, they assessed their own performance using the same sheet as the evaluator’s and completed the survey for emotional response and self-efficacy. Chi-squared tests, Fisher’s exact tests, independent sample Student’s *t* tests, and Mann–Whitney nonparametric *U* tests, and Analysis of covariance (ANCOVA) were used to compare the baseline measurements of the demographic characteristics and the dependent variables between the PF and NF groups.

**Results:**

The NF group demonstrated a more accurate self-rated assessment than the PF group (*p* <  0.001). While self-efficacy (*p* <  0.001) and positive emotions (*p* <  0.001) were significantly stronger in the PF group than in the NF group, negative emotions were significantly stronger in the NF group than in the PF group (*p* = 0.001).

**Conclusions:**

Evaluator’s verbal feedback exerts a significant influence on the accuracy of self-assessment as well as emotions and self-efficacy. Instructors should pay attention to providing feedback to students, taking into account the impact of positive or negative feedback.

## Background

Feedback can enhance the student’s ability to perform a task and judge their own performance [1]. Van De Ridder et al. [2] described feedback as “being specific information about the comparison between a trainee’s observed performance and a standard, given with the intent to improve performance”. Students are given the opportunity for feedback to determine any performance gaps and to improve performance areas in which they might be expected to self-assess accurately. Self-assessment ability is one of the key elements of self-directed and performance-based learning, there is a need to measure students’ abilities to accurately self-assess their own performance. However, a review on the literature of self-assessment compared with observed measures raised concerns about one’s limited ability to self-assess accurately [3]. A feedback strategy is therefore needed to reduce the gap and enhance the appropriateness of self- assessment.

Feedback can be categorized as positive or negative. Positive feedback is used to indicate that an expected or desired behavior was demonstrated, or to reinforce successive steps toward a goal. Negative feedback indicates that a behavior or task was not performed correctly, thus indicating that a change of behavior is needed [4]. It has been found generally that those who receive positive feedback achieve greater success in subsequent performance while those who receive negative feedback perform worse [5]. However, some studies have reported contrary findings in which constructive criticism is more effective at improving skill than compliments [6, 7]. The findings of studies are inconsistent as to which type of feedback helps students to improve their performance or the ability to judge their own performance.

Feedback can lead to positive or negative emotional reactions [8]. It is increasingly important to accommodate the emotional response of students receiving feedback [9]. As the feedback delivery from instructors or supervisors causes emotional responses in the recipients, these can affect not only performance but also lead to counterproductive behaviors [10]. The affective process through which individuals interpret performance feedback is also an important mechanism for explaining behavioral self-regulation [11]. However, the emotional consequences of such feedback have been overlooked [12]. In healthcare education, corrective and constructive feedback is often used to improve performance, it is necessary to recognize the impact of feedback on emotions.

Feelings of self-efficacy are important mediators in feedback situations [13]. Self-efficacy is a self-appraisal of one’s ability to master a task, which includes judgments about one’s capability to fulfill given task demands and to use feedback as needed to attain a goal [14]. It is shaped by self-beliefs about one’s skills within a specific context [12]. Self-efficacy is considered to be a significant variable in student learning, because it affects students’ motivation and learning [15]. Kluger and DeNisi [16] state that feedback is effective to the degree to which it directs information to enhance self-efficacy and to more effective self-regulation, such that attention is directed back to the task and causes students to invest more effort or commitment to the task. However, little is known about the relationship between instructor’s feedback and self-efficacy for learning and performance in skill training. A few studies outside the healthcare education have shown that performance feedback has an impact on domain-specific self-efficacy [17]. Negative feedback diminished self-efficacy while positive feedback was related to higher level of self-efficacy [17]. Positive feedback could have favorable effects on motivation and self-efficacy, which has been suggested to be positively associated with improvements in performance [11]. At this time, it is important to distinguish between praise that has low information value regarding achievement and learning, and praise directed to effort, self-regulation, engagement, or processes relating to the task when giving positive feedback to enhance self-efficacy [13].

Most studies on feedback are limited to focusing on investigating the recipients’ performance improvement rather than the appropriateness of self-rated assessment or affective aspects. Also, there is both supporting and contradictory evidence regarding the effectiveness of positive and negative feedback in changing behaviors and facilitating learning. This study examined the effects of an examiner’s positive and negative verbal feedback on the accuracy of self-assessment, emotional responses, and self-efficacy for learning and performance in the skill performance assessment.

## Methods

### Design

This study employed a quasi-experimental posttest design to evaluate the effects of examiner’s positive and negative verbal feedback on the accuracy of self-assessment, emotional responses, and self-efficacy.

### Participants and setting

This study was approved by the ethics committee of Hallym University. The sample size was calculated for independent sample *t* tests between positive feedback (PF) and negative feedback (NF) groups using G Power 3.0.10. The probability for a Type I error (alpha) was set at 0.05, the power (1–beta) was set at 0.8, and the medium effect size was set at 0.5. The required sample size was 51 for each group. In general, the baccalaureate nursing curriculum in South Korea is similar to those of bachelor of science in nursing (BSN) in United States. Eligible participants were all second-year nursing students who taking a course of the fundamental nursing at a university in South Korea. All 111 students agreed to participate in the study. Following an informed consent procedure, students were assigned randomly to the PF group (*n* = 58) or the NF group (*n* = 53). One student in the control group dropped out because of missing data in the questionnaire, the final subjects were 110 students, 58 in the PF group and 52 in the NF group.

### Measures

First, the instructor rated the student’s skill performance by observing using a checklist from the protocol of the Korean Accreditation Board of Nursing Education [18] applying rigorous step-by-step procedures. In the checklist, patient identification, explanation of the purpose of the procedure, details of critical procedural elements, and cleaning up were listed in that order. A dichotomous scoring scale of 0 = not done/done incorrectly and 1 = done correctly was imposed for each item.

Following completion of the skill performance and feedback, students completed a self-report questionnaire, which included measures of self-rated one’s skill performance, emotional responses, self-efficacy, and general characteristics such as age, gender, and grade point average (GPA) for previous academic achievement.

The accuracy of self-assessment of performance was calculated for each subject as the difference between the observed actual score and the student’s own self-rated score.

Emotional responses were measured by utilizing Warr’s questionnaire items [19] to identify the emotional differences of participants depending on the types of feedback they were given. In this study, a total of 10 types of emotion were selected that can appear during skill performance testing: five positive (cheerful, glad, contented, comfortable, or relaxed) and five negative (unsatisfied, anxious, tense, sad, or discouraged) emotion terms. A 5-point Likert scale (1 = strongly disagree to 5 = strongly agree) was used with this instrument. Cronbach’s alpha coefficients of these positive and negative response scales were 0.89 and 0.83 respectively.

Self-efficacy was measured with a self-efficacy subscale of the Motivated Strategies for Learning Questionnaire developed by Pintrich et al. [20]. The questionnaire consists of eight items. A 7-point Likert scale (1 = strongly disagree to 7 = strongly agree) was used with this instrument. The sum of the item scores reflects self-efficacy for learning and performance. A higher score indicates a higher level of self-efficacy of the respondent. The reported Cronbach’s alpha coefficient of this scale was 0.93 in Pintrich et al. [20] and 0.94 in this study.

### Procedures

Students were randomly assigned a type of feedback (positive or negative) using a simple random extraction lot, and they were not informed of the type of feedback they would receive. The instructor assessed the student’s performance with a prepared evaluation sheet, did not provide any feedback during the performance evaluation and maintained verbal and nonverbal neutrality. After completing the skill performance, each student received a predetermined type of verbal feedback regardless of whether he or she did well or not from one of the two instructors who had been observing their performance on a one-to-one basis. Even though the student was very good at performing, there was still room for improvement, so it was not a problem even if the student was placed in negative feedback. Conversely, if a student who was assigned to positive feedback did not perform well, he received positive feedback that focused on what was good. The instructor provided behavior-based feedback in a neutral manner following the guidelines for the two types of feedback that were developed in advance.

The positive feedback consisted of general compliments (e.g., “Great!” or “Well done”) and then three specific positive comments about his/her performance. The negative feedback consisted of general criticisms (e.g., “More effort required” or “Wrong”) and then three specific constructive comments about his/her performance. After receiving the feedback, the students assessed their own performance with the same evaluation sheet. The students then completed a survey of their perceptions of emotional response and self-efficacy. At the completion of the session, the instructor provided correct feedback to students whose received feedback was not consistent with their true performance. For example, a student who received only positive feedback despite the performance requiring significant remediation (having made many mistakes) was given an opportunity to correct the survey later. Data collection of this study was conducted throughout the day in a nursing laboratory in the school.

### Data analysis

Shapiro–Wilk tests were conducted to assess the normal distribution of all variables. Chi-squared tests, Fisher’s exact tests, independent sample Student’s *t* tests, and Mann–Whitney nonparametric *U* tests were used to compare the baseline measurements of the demographic characteristics and the dependent variables between the PF and NF groups. Analysis of covariance (ANCOVA) was used to compare mean differences between the groups, in which age and performance test scores were used as covariates to control for differences in baseline characteristics. At that point, data that were not normally distributed were adjusted with natural log transformation. Data were analyzed using IBM SPSS Statistics 21.0 and the level of significance was set at *p* <  0.05 in two-tailed tests.

## Results

### Baseline characteristics and homogeneity test

The baseline characteristics of the students are listed in Table 1. There were no significant differences in gender or GPA for previous academic achievement between the two groups. However, there were significant differences between the groups in age and performance test scores. The median ages in the PF and NF groups were 20 and 20 years, which was significantly different (Mann–Whitney *U* = 1170.0, *p* = 0.026). The median performance test scores in the PF and NF groups were 36 and 35, respectively, which was statistically significant (Mann–Whitney *U* = 693.0, *p* <  0.001).

**Table 1:** Homogeneity of general characteristics between groups

Variables	Categories		PF group (*n* = 58)	NF group (*n* = 52)	*U* or χ^2^	*p*
Age (years)		Median (min, max)	20.0 (19, 24)	20.0 (19, 23)	1177.0	0.026
		M ± SD	20.8 ± 1.2	20.3 ± 0.8		
Gender	Male		15 (25.9)	11 (21.2)	0.337	0.655*
	Female		43 (74.1)	41 (78.8)		
GPA for previous academic achievement		Median (min, max)	3.8 (0.6, 4.4)	3.6 (1.9, 4.5)	1491.0	0.919
		Mean ± SD	3.6 ± 0.7	3.6 ± 0.6		
Performance test score		Median (min, max)	36.0 (22, 38)	35 (26, 38)	693.0	< 0.001
		M ± SD	34.9 ± 3.6	34.2 ± 2.6		

*Fisher’s exact test

*GPA* grade point average, *PF* positive feedback, *NF* negative feedback, *SD* standard deviation

### Effects of feedback on accuracy of self-assessment

The self-assessment scores and the accuracy of self-assessment are presented in Table 2. The students’ mean and standard deviation self-assessment scores in the PF and NF groups were 36.8 ± 1.8 (median 37) and 35.0 ± 2.0 (median 35), respectively (ANCOVA F = 18.37, *p* < 0.001). The accuracy of self-assessment is the difference between the self-assessment score and actual score. A value closer to zero means that the self-assessment is more accurate. The mean difference between the self-assessment score and actual score in the PF group was 2.1 ± 3.2 (median = 1), which was higher than the difference in the NF group of 0.8 ± 1.9 (median = 1; ANCOVA F = 20.64, *p* < 0.001).

**Table 2:** Comparison of groups for self-evaluated score and accuracy of self-evaluation

Variables		PF group (*n* = 58)	NF group (*n* = 52)	*U*	*p*	ANCOVA F (*p*)
Self-assessment score	Median (min, max)	37.0 (27, 38)	35.0 (30, 38)	693.0	< 0.001	18.37 (< 0.001)
	Mean ± SD	36.8 ± 1.8	35.0 ± 2.0			
Difference (self-test score)	Median (min, max)	1.0 (−3, 14)	1.0 (−2, 7)	1167.0	0.039	20.64 (< 0.001)
	M ± SD	2.1 ± 3.2	0.8 ± 1.9			

ANCOVA was performed to examine the differences in dependent variables between the two groups after covariance treatment of unequal age and scores in the baseline characteristics. In this case, variables that did not satisfy the assumption of normality were transformed to natural logarithms

*PF* positive feedback, *NF* negative feedback

### Effects of feedback on emotional response and self-efficacy

The scores of emotional response and self-efficacy in the PF and NF groups are presented in Table 3. The mean positive emotion score in the PF group was 14.3 ± 4.9 (median 14), which was higher than the score of 9.6 ± 3.7 (median 9) in the NF group (ANCOVA F = 20.93, *p* < 0.001). The mean negative emotion score in the PF group was 12.1 ± 4.0 (median 12), which was lower than the score of 15.2 ± 4.3 (median 15) in the NF group (ANCOVA F = 12.77, *p* = 0.001). The mean self-efficacy score for the PF group was 42.0 ± 8.3, which was higher than the score of 37.8 ± 7.3 (ANCOVA F = 5.16, *p* = 0.025) in the NF group.

**Table 3:** Comparison of groups for emotional response and self-efficacy

Variables		PF group (*n* = 58)	NF group (*n* = 52)	*t* or *U*	*p*	ANCOVA F (*p*)
Positive emotion	Median (min, max)	14.0 (5, 25)	9.0 (5, 18)	523.0	0.000	20.93 (< 0.001)
	Mean ± SD	14.3 ± 4.9	9.6 ± 3.7			
Negative emotion	Median (min, max)	12.0 (6, 24)	15.0 (8, 25)	665.0	0.000	12.77 (0.001)
	Mean ± SD	12.1 ± 4.0	15.2 ± 4.3			
Self-efficacy	Mean ± SD	42.0 ± 8.3	37.8 ± 7.3	2.595	0.011	5.16 (0.025)

ANCOVA was performed to examine the differences in dependent variables between the two groups after covariance treatment of unequal age and scores in the baseline characteristics. In this case, variables that did not satisfy the assumption of normality were subjected to normal conversion processing with natural logarithm

*PF* positive feedback, *NF* negative feedback

## Discussion

In this study, the type of feedback provided to students was associated with the accuracy of self-assessment as well as emotional responses and self-efficacy. As expected, negative feedback from examiner was associated with more accurate assessment of one’s own skill performance than positive feedback. This result is consistent with the findings of Plakht et al. [5] in which high-quality negative feedback was linked to an accurate self-evaluation of students’ performance in clinical practice, whereas high-quality positive feedback was associated with self-overestimated performance. It seems that negative feedback helps students to assess their performance more realistically and accurately than positive feedback. In our study, students’ self-assessment scores were higher than the actual scores in both the PF and NF groups and the level of overestimation was significantly higher in the former group. A possible explanation for these findings concerning the PF group is that positive feedback influenced students into experiencing a happier state of mind and higher self-efficacy feelings, which led them to evaluate their performance at a much higher level. This is in accordance with the findings of Papinczak et al. [21] in which students with higher self-efficacy feelings evaluated their performance at a much higher level than did those with feelings of low self-efficacy. Positive feedback often causes a positive emotional reaction, which has been associated with increased motivation [9]. Indeed, the impact of evaluative feedback on participants’ emotional positivity and self-efficacy was higher in the PF group than in the NF group. By contrast, those who received negative feedback might become discouraged and lose confidence. These findings from the current study are largely in line with those of many studies showing that positive feedback significantly increases self-efficacy and/or that negative feedback has a significant negative effect on self-efficacy [17, 21–23]. The feeling of self-efficacy differed according to the type of feedback given; that is, self-efficacy refers to individuals’ expectations and assurance of what they can achieve in a given situation [24]. By this very interaction, self-efficacy is considered to depend on context. These results mean that the type of feedback might be linked to changing motivation to learn within the context of nursing skills development. For most students, positive feedback is perceived to be a motivator to practice. This is in accordance with the findings of Kannappan et al. [7] in which positive feedback was clearly more likely to lead to a desire to learn. In addition, positive feedback was associated with better performance of students in clinical practice, as the students delivered better care and thus received better grades [5, 21]. In the field of education, Shute [25] identified a negative correlation between negative feedback and self-esteem, which eventually lowered learning performance.

In our study, because the performance measure was an important task reflected in the academic achievement, examiner’s feedback after completion of the task would have had a significant impact on the student’s emotional reaction and self-efficacy. In the previous study, because the result of the task performance was not reflected in the actual school records of the students, the positive or negative feedback given to the students did not affect leaners’ emotion and perceived competence [26]. These results indicate that the evaluator should consider students’ emotional aspects and feeling of self-efficacy when providing performance feedback, especially in high-stakes assessment.

Given the nature of our study, implications need to be considered with caution. Negative feedback was associated with more accurate self-assessment of performance than was positive feedback, which supports previous research that suggested that a constructive feedback can enhance the students’ ability to reflect on themselves more accurately [27]. However, we found that positive feedback produced stronger positive emotions and higher self-efficacy than did negative feedback. Although students’ affective component of evaluative feedback from their instructor might not be an indicator of the quality of feedback or of key function of feedback, it should not be overlooked as a learning motivator. Students should not lose confidence from experiencing negative emotions while acquiring skills. Performance feedback should include elements of compliments and behavior that encourage change in a balanced way, as this combination will enhance educational and emotional outcomes.

### Limitations

Our study had several limitations. First, the results cannot be generalized because this study was conducted within one nursing course at a single university. Second, the relationship between instructor and student has already been formed, so it is possible that even though feedback was given according to the study protocol, the relationship between the student and the instructor might have influenced the student’s reactions. Nevertheless, this research is distinctive in that it empirically investigated the difference between self-assessment and actual assessment scores, emotional responses, and motivation overall following positive and negative feedback while acquiring key nursing skills.

## Conclusions

This study demonstrated that negative feedback provided students the opportunity of more accurate self-assessment, but also produced negative emotional responses and less self-efficacy. It is necessary to explore the practical implications of these findings, because it was found that verbal feedback significantly impacted individuals’ self-efficacy and emotional response as well as the accuracy of self-assessment. Thus, educators who are responsible for teaching and assessing students’ performance should utilize feedback in a productive way. Ultimately, a more useful approach would be to focus on reducing the variation between self-rated and actual objective assessments simultaneously, to enhance learning motivation in an emotionally safe environment. Future research should assess the relationship between the type of feedback and the perceived quality of feedback. In addition, the type of feedback that simultaneously meets educational outcomes and emotional safety should be studied.

## Abbreviations

ANCOVA: Analysis of covariance
GPA: Grade point average
NF: Negative feedback
PF: Positive feedback
SD: Standard deviation

## References

[1] Fotheringham D (2011). The role of expert judgement and feedback in sustainable assessment: a discussion paper. Nurse Educ Today.

[2] Van de Ridder JM, Stokking KM, McGaghie WC, Ten Cate OT (2008). What is feedback in clinical education?. Med Educ.

[3] Davis DA, Mazmanian PE, Fordis M, Van Harrison RTKE, Thorpe KE, Perrier L (2006). Accuracy of physician self-assessment compared with observed measures of competence: a systematic review. JAMA..

[4] Sprouls K, Mathur SR, Upreti G (2015). Is positive feedback a forgotten classroom practice? Findings and implications for at-risk students. Prev Sch Fail.

[5] Plakht Y, Shiyovich A, Nusbaum L, Raizer H (2013). The association of positive and negative feedback with clinical performance, self-evaluation and practice contribution of nursing students. Nurse Educ Today.

[6] Boehler ML, Rogers DA, Schwind CJ, Mayforth R, Quin J, Williams RG (2006). An investigation of medical students reactions to feedback: a randomized controlled trial. Med Educ.

[7] Kannappan A, Yip DT, Lodhia NA, Morton J, Lau JN (2012). The effect of positive and negative verbal feedback on surgical skills performance and motivation. J Surg Educ.

[8] Ilgen DR, Davis CA (2000). Bearing bad news: reactions to negative performance feedback. Appl Psychol.

[9] Archer JC (2010). State of the science in health professional education: effective feedback. Med Educ.

[10] Belschak FD, Den Hartog DN (2009). Consequences of positive and negative feedback: the impact on emotions and extra-role behaviors. Appl Psychol.

[11] Ilies R, De Pater IE, Judge T (2007). Differential affective reactions to negative and positive feedback, and the role of self-esteem. J Manag Psychol.

[12] Brockner J, Higgins ET (2001). Regulatory focus theory: implications for the study of emotions at work. Organ Behav Hum Decis Process.

[13] Hattie J, Timperley H (2007). The power of feedback. Rev Educ Res.

[14] Zimmerman BJ (2000). Self-efficacy: an essential motive to learn. Contemp Educ Psychol.

[15] van Dinther M, Dochy F, Segers M (2011). Factors affecting students’ self-efficacy in higher education. Educ Res Rev.

[16] Kluger AN, DeNisi A (1996). The effects of feedback interventions on performance: a historical review, a meta-analysis, and a preliminary feedback intervention theory. Psychol Bull.

[17] Daniels JA, Larson LM (2001). The impact of performance feedback on counseling self-efficacy and counselor anxiety. Couns Educ Superv.

[18] Korean Accreditation Board of Nursing Education. Accreditation criteria of nursing education. 2013. http://www.kabone.or.kr/kabon02/index04.php. Accessed 23 Mar 2015.

[19] Warr PB, Bindl U, Parker SK, Inceoglu I (2014). Four-quadrant investigation of job-related affects and behaviours. Eur J Work Organ Psychol.

[20] Pintrich PR, Smith DA, Garcia T, McKeachie WJ (1993). Reliability and predictive validity of the motivated strategies for learning questionnaire (MSLQ). Educ Psychol Meas.

[21] Papinczak T, Young L, Groves M, Haynes M (2007). An analysis of peer, self, and tutor assessment in problem-based learning tutorials. Med Teach..

[22] Reynolds D (2006). To what extent does performance-related feedback affect managers' self-efficacy?. Int J Hosp Manag.

[23] Saemi E, Porter JM, Ghotbi-Varzaneh A, Zarghami M, Maleki F (2012). Knowledge of results after relatively good trials enhances self-efficacy and motor learning. Psychol Sport Exerc.

[24] Bong M, Skaalvik EM (2003). Academic self-concept and self-efficacy: how different are they really?. Educ Psychol Rev.

[25] Shute VJ (2008). Focus on formative feedback. Rev Educ Res.

[26] Jin S (2016). Effects of motivational dispositions and feedback types on learners' emotions with the unfamiliar task. J Learner-Centered Curriculum Instruction.

[27] Colthart I, Bagnall G, Evans A, Allbutt H, Haig A, Illing J (2008). The effectiveness of self-assessment on the identification of learner needs, learner activity, and impact on clinical practice: BEME guide no. 10. Med Teach.

